# Magnetic resonance imaging-based detection of retinal hemorrhages in a multicenter cohort of abusive head trauma

**DOI:** 10.1007/s00247-026-06558-6

**Published:** 2026-03-04

**Authors:** Maria Hahnemann, Bernd Karger, Sibylle Banaschak, Hans-Joachim Mentzel, Alexander Radbruch, Daniel Wittschieber

**Affiliations:** 1 Department of Diagnostic and Interventional Neuroradiology and Pediatric Neuroradiology, University Hospital Bonn, University of Bonn, Venusberg-Campus 1, 53127 Bonn, Germany; 2 Institute of Legal Medicine, University Hospital Münster, University of Münster, Röntgenstraße 23, 48149 Münster, Germany; 3 University of Cologne, Faculty of Medicine and University Hospital Cologne, Institute of Legal Medicine, Melatenguertel 60/62, 50823 Cologne, Germany; 4 Department of Radiology, Section of Pediatric Radiology, Jena University Hospital, Friedrich Schiller University Jena, Am Klinikum 1, 07747 Jena, Germany; 5 Institute of Forensic Medicine, University Hospital Bonn, University of Bonn, Stiftsplatz 12, 53111 Bonn, Germany

**Keywords:** Abusive head trauma, Child physical abuse, Magnetic resonance imaging, Non-accidental head injury, Retinal hemorrhage, Shaken baby syndrome

## Abstract

**Background:**

In pediatric abusive head trauma (AHT), retinal hemorrhages are a key diagnostic feature. Detection by fundoscopy may be delayed or limited, whereas magnetic resonance imaging (MRI) enables non-invasive, objective assessment on routine brain sequences.

**Objective:**

To evaluate the diagnostic utility of different MRI sequences—particularly susceptibility-weighted imaging (SWI), T2*-weighted (T2*w), and morphological sequences—in detecting retinal hemorrhages in AHT.

**Materials and methods:**

In this retrospective multicenter study (2006–2015), 57 well-documented AHT cases from three German institutions were analyzed. A subgroup consisted of “confession cases.” MRI scans were reviewed for retinal hemorrhages across SWI, T2*w, T1-weighted, T2-weighted (T2w), and fluid-attenuated inversion recovery (FLAIR) sequences by blinded expert readers. Fundoscopy results served as the gold standard. Sensitivities were calculated for each sequence, and “confession” versus “non-confession” cases were compared.

**Results:**

Fundoscopy detected retinal hemorrhages in 44 of 56 evaluable cases (78.6%). MRI identified retinal hemorrhages most frequently on gradient recalled echo sequences, with SWI showing higher sensitivity compared to T2*w (76.9% vs. 47.8%). T2w imaging showed markedly lower sensitivity (30.3%) but detected retinal hemorrhages on one eye missed on T2*w imaging in two cases. In three cases, MRI detected retinal hemorrhages not reported on fundoscopy. No statistically significant differences were found between “confession” and “non-confession” cases across all parameters considered (*P*>0.05).

**Conclusion:**

MRI—particularly SWI and, to a lesser extent, T2*w imaging—may offer a useful tool of detecting retinal hemorrhages in AHT when fundoscopy is limited. T2w imaging may provide complementary information in selected cases.

**Graphical abstract:**

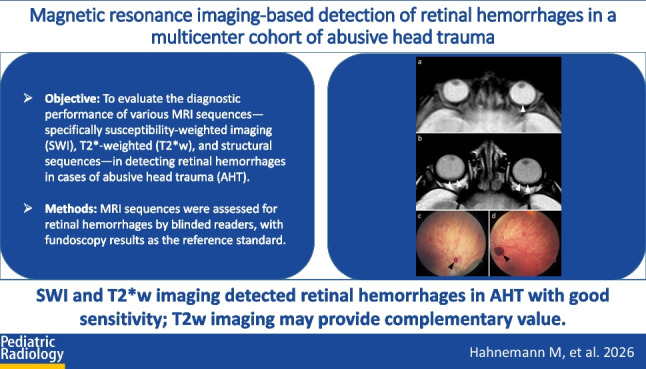

## Introduction

Abusive head trauma (AHT) is a leading cause of morbidity and mortality in infants and young children [1]. The term AHT includes various mechanisms such as violent shaking, direct blunt force impacts, or combinations thereof [2]. Its presentation is non-specific and may be subtle, making clinical assessment difficult and frequently delaying diagnosis [2, 3]. Such delays can adversely affect clinical outcomes and medico-legal investigations, increasing the risk of recurrent abuse. Therefore, improving AHT diagnostics would be of clinical and forensic importance.

Besides subdural collections and injuries of the brain parenchyma [4, 5], the presence of retinal hemorrhages has an evidential value towards AHT following violent shaking [6, 7]. Although neither obligatory nor specific, retinal hemorrhages are present in a substantial majority of suspected cases and play a pivotal role in distinguishing AHT from accidental head trauma. Retinal hemorrhages were described in about 80% (45–100%) of AHT cases, typically exhibiting greater severity, multilayer distribution, and involvement of the peripheral retina [6, 8–11]. The pathophysiology of retinal hemorrhages in this context involves vitreoretinal traction resulting from repetitive acceleration-deceleration forces, a mechanism less prominent in accidental trauma [10, 12, 13].

The established reference for the detection of retinal hemorrhages in infants with suspected AHT is a fundoscopy performed by an ophthalmologist under pupillary dilation, preferably using indirect ophthalmoscopy [10]. Moreover, wide-field digital retinal imaging systems such as RetCam enable the retention of examination images and review by additional practitioners, both of which may be important for gathering evidence in cases of suspected abusive head trauma [14]. However, young children with altered mental status or severe trauma are difficult to examine, and technical limitations, such as incomplete visualization of the peripheral retina, can impede a comprehensive assessment [10, 15]. These issues can diminish diagnostic yield, increase interobserver variability, and delay diagnosis.

The implementation of gradient recalled echo (GRE) sequences as T2*-weighted (T2*w) imaging and susceptibility-weighted imaging (SWI) in magnetic resonance imaging (MRI) has opened the possibility of non-invasive detection of retinal hemorrhages interpretable by radiologists. Several different studies have already demonstrated the utility of MRI, especially GRE for the detection of retinal hemorrhages in AHT [16–19]. MRI also has the potential to visualize retinal hemorrhages in a more objective and less examiner-dependent manner, thereby supporting the diagnosis of AHT and providing documentation that may be useful in medico-legal expert reports and criminal proceedings.

Although initial data regarding the diagnostic capability of MRI for detecting retinal hemorrhages are encouraging [16–20], the current body of evidence is still limited by small sample sizes, heterogeneous study designs, and methodological variability. Consequently, further studies in well-defined and methodologically robust cohorts are needed to substantiate previous results and to delineate the diagnostic utility of MRI more precisely. An additional diagnostic tool for the detection and documentation of retinal hemorrhages would be desirable, especially in situations where fundoscopy is unavailable or can only be performed with delay. The aim of this study is therefore to assess the diagnostic concordance between MRI-detected retinal hemorrhages and those identified via fundoscopy across various MRI sequences.

## Materials and methods

### Data collection

This retrospective, multicenter study uses a study cohort that was compiled at the Institutes of Legal Medicine at the Universities of the German cities of Cologne, Essen, and Münster, encompassing the period from 2006 to 2015. The study was approved by the institutional review boards of all participating institutions (University Hospitals of Cologne, Essen, and Münster; no. 2014-658-f-N, Medical Association of Westfalen-Lippe and the Westphalian Wilhelms University). Due to the study design, informed consent of the study subjects and their relatives was waived.

The original cohort consisted of 72 cases of AHT, as previously reported [5, 21–26]. The diagnosis of AHT was established through the consensus of a multi-headed clinical/medico-legal team based on a comprehensive diagnostic evaluation. In all cases, the explicit diagnosis of “shaken baby syndrome” was made so that the major trauma mechanism of this AHT cohort is assumed to be violent shaking. As reported before [23], the presence of a perpetrator’s confession in 15 cases allowed a further stratification of the study cohort into “confession cases” (cases with confessed perpetrators) and “non-confession cases” (cases without confessed perpetrators). The comparison between these two subgroups reinforces diagnostic validity and mitigates the potential for circular bias.

### Neuroimaging

For this study, all cases with MR neuroimaging performed within 14 days after admission to hospital were included for analysis (*n*=57). Imaging techniques and protocols varied due to the extended study period and the retrospective multicenter study design. MRI was obtained on either a 1.5-T (*n*=47) or 3-T scanner (*n*=10). Standard MRI protocols essentially included T1-weighted (T1w), T2-weighted (T2w), fluid-attenuated inversion recovery (FLAIR), and GRE (T2*w and/or SWI) imaging. The imaging protocols included the following acquisition parameters: T1w images had a repetition time (TR) of 244–1000 ms, echo time (TE) of 5–15 ms, slice thickness (ST) of 3–6 mm, and a slice gap (SG) of 3.3–6.5 mm. T2w images had a TR/TE of 3,000–10,000/81–160 ms, ST of 2–5.5 mm, and a SG of 1–6.5 mm. FLAIR images had a TR/TE of 5,700–11,000/80–140 ms, ST of 2–6 mm, and a SG of 1.2–6.6 mm. T2*w images had a TR/TE of 400–2,200/15–40 ms, ST of 2–7, and a SG of 1.2–8.4 mm. SWI had a TR/TE of 28–51/20–40 ms and a ST of 1.2–3 mm. Field of view ranged between 150–230 mm.

### Image analysis

MRI evaluation was jointly conducted by a board-certified radiologist (M.H.) and a board-certified forensic physician (D.W.), each with over 10 years of specialized experience in the neuroimaging of child abuse cases. All available images were qualitatively assessed for the presence or absence of retinal hemorrhages. The two readers were blinded to the results of the ophthalmological examination. Retinal hemorrhages were defined as areas of low signal intensity along the retina. To enhance comparability, image analysis was limited to the axial plane. For subsequent analysis, MRI findings were evaluated on a per eye basis in comparison with the fundoscopy.

### Ophthalmological assessment

Based on previous analyses of the available medical records [21], which included written documentations of fundoscopy, the results of these assessments were collected and reviewed. Retinal hemorrhages were documented as either present or absent, without any grading of severity. Moreover, the interval between ophthalmological assessment and hospital admission was recorded. Fundoscopy was considered the gold standard.

### Statistical analysis

Statistical data analyses were performed using IBM SPSS Statistics (version 29.0.0.0) (IBM Corporation, Armonk, NY). Continuous variables were presented as the mean±standard deviation, median, and range and compared using *t* test. Categorical variables were assessed by the Fisher exact test. *P*-values of less than 0.05 were considered statistically significant.

## Results

A total of 57 children were included in the study: 38 were male and 19 were female, with a mean age of 4.1 months±3.7 months standard deviation (range 0–17 months, *n*=56/57 with age≥1 month). The comparison between “confession cases” and “non-confession cases” did not show any statistically significant difference regarding sex (*P*=1.000) and age (*P*=0.059).

Fundoscopy was available in 98.2% of cases (56/57); in all of these cases, both eyes were evaluable. Retinal hemorrhages were identified in 78.6% of the cases (44/56), with retinal hemorrhages on a per-eye basis of 71.4% (80/112).

In all cases with available fundoscopy, both eyes were evaluable in the existing MRI sequences. A summary of the MRI sequences available for all cases with fundoscopy is provided in Table 1. For eyes with retinal hemorrhages confirmed on fundoscopy, MRI demonstrated retinal hemorrhages in 22/46 eyes (47.8%) on T2*w imaging, 20/26 eyes (76.9%) on SWI, 1/65 eyes (1.5%) on T1w imaging, 20/66 eyes (30.3%) on T2w imaging, and 2/46 eyes (4.3%) on FLAIR imaging. False-positive findings occurred in three eyes on T2*w imaging, one eye on SWI, two eyes on T2w imaging, and one eye on FLAIR imaging, while no false positives were observed on T1w imaging. A summary of the fundoscopy and MRI findings for all 56 cases with available MRI and fundoscopy data is provided in Table 2.

**Table 1:** Overview of imaging availability of different magnetic resonance imaging (*MRI*) sequences

Imaging	Availability in fundoscopy, available cases (%)
MRI	56/56 (100%)
T2*w	36/56 (64.3%)
SWI	17/56 (30.4%)
T1w	44/56 (78.6%)
T2w	47/56 (83.9%)
FLAIR	35/56 (62.5%)

*%* percent, *FLAIR* fluid-attenuated inversion recovery, *n* number of cases, *SWI* susceptibility-weighted imaging, *T1w* T1-weighted, *T2w* T2-weighted, T2*w T2*-weighted

**Table 2:** Retinal hemorrhage detection in fundoscopy and magnetic resonance imaging (*MRI*) sequences, reported separately for the right and left eyes, comprising all cases with both MRI and fundoscopy data available

Case number	Fundoscopy		T2*w		SWI		T1w		T2w		FLAIR	
	**R**	**L**	**R**	**L**	**R**	**L**	**R**	**L**	**R**	**L**	**R**	**L**
**1**	** + **	**–**	NA	NA	NA	NA	**–**	**–**	**–**	**–**	**–**	**–**
**2**	** + **	** + **	** + **	** + **	NA	NA	**–**	**–**	**–**	** + **	**–**	**–**
**3**	**–**	**–**	**–**	**–**	NA	NA	**–**	**–**	**–**	**–**	**–**	**–**
**4**	** + **	** + **	**–**	**–**	NA	NA	NA	NA	**–**	**–**	NA	NA
**5**	** + **	**–**	**–**	**–**	NA	NA	NA	NA	**–**	**–**	NA	NA
**6**	** + **	** + **	NA	NA	** + **	** + **	**–**	**–**	** + **	** + **	**–**	**–**
**7**	** + **	** + **	+	+	** + **	** + **	**–**	**–**	** + **	** + **	NA	NA
**8**	** + **	** + **	**–**	**–**	NA	NA	**–**	**–**	**–**	**–**	NA	NA
**9**	**–**	**–**	**–**	**–**	NA	NA	NA	NA	**–**	**–**	**–**	**–**
**10**	** + **	** + **	** + **	** + **	NA	NA	NA	NA	**–**	**–**	**–**	**–**
**11**	** + **	** + **	**–**	** + **	NA	NA	**–**	**–**	** + **	** + **	NA	NA
**12**	**–**	**–**	**–**	**–**	NA	NA	**–**	**–**	**–**	**–**	**–**	**–**
**13**	** + **	** + **	** + **	** + **	NA	NA	**–**	**–**	** + **	** + **	NA	NA
**14**	** + **	** + **	**–**	**–**	NA	NA	NA	NA	**–**	**–**	NA	NA
**15**	** + **	** + **	+	+	** + **	** + **	**–**	**–**	**–**	**–**	**–**	**–**
**16**	** + **	** + **	NA	NA	**–**	**–**	**–**	**–**	**–**	**–**	**–**	**–**
**17**	**–**	**–**	**–**	**–**	**–**	**–**	**–**	**–**	**–**	**–**	NA	NA
**18**	** + **	**–**	NA	NA	** + **	**–**	**–**	**–**	**–**	**–**	**–**	**–**
**19**	** + **	** + **	NA	NA	** + **	** + **	** + **	**–**	** + **	** + **	** + **	** + **
**20**	** + **	** + **	**–**	**–**	NA	NA	NA	NA	**–**	**–**	**–**	**–**
**21**	** + **	** + **	** + **	** + **	NA	NA	**–**	**–**	** + **	**–**	**–**	**–**
**22**	**–**	+	NA	NA	NA	NA	NA	NA	**–**	**–**	NA	NA
**23**	** + **	** + **	**–**	**–**	NA	NA	**–**	**–**	NA	NA	**–**	**–**
**24**	** + **	** + **	**–**	**–**	NA	NA	**–**	**–**	**–**	**–**	NA	NA
**25**	** + **	** + **	** + **	** + **	NA	NA	**–**	**–**	** + **	** + **	NA	NA
**26**	** + **	** + **	NA	NA	**–**	**–**	**–**	**–**	**–**	**–**	NA	NA
**27**	** + **	**–**	**–**	**–**	NA	NA	NA	NA	NA	NA	**–**	**–**
**28**	** + **	** + **	NA	NA	NA	NA	**–**	**–**	NA	NA	NA	NA
**29**	** + **	** + **	** + **	**–**	NA	NA	**–**	**–**	** + **	** + **	**–**	**–**
**30**	**–**	** + **	**–**	** + **	NA	NA	**–**	**–**	**–**	**–**	**–**	**–**
**31**	**–**	**–**	** + **	** + **	NA	NA	NA	NA	**–**	** + **	**–**	** + **
**32**	** + **	** + **	NA	NA	NA	NA	**–**	**–**	**–**	**–**	NA	NA
**33**	**–**	**–**	**–**	**–**	NA	NA	**–**	**–**	**–**	**–**	**–**	**–**
**34**	** + **	** + **	**–**	**–**	NA	NA	**–**	**–**	**–**	**–**	**–**	**–**
**35**	**–**	**–**	**–**	**–**	**–**	**–**	NA	NA	NA	NA	**–**	**–**
**36**	** + **	** + **	NA	NA	NA	NA	**–**	**–**	**–**	**–**	**–**	**–**
**37**	** + **	** + **	NA	NA	NA	NA	**–**	**–**	** + **	**–**	NA	NA
**38**	**–**	**–**	**–**	**–**	NA	NA	**–**	**–**	**–**	**–**	**–**	**–**
**39**	** + **	** + **	**–**	**–**	NA	NA	**–**	**–**	**–**	**–**	**–**	**–**
**40**	** + **	** + **	**–**	**–**	NA	NA	**–**	**–**	**–**	**–**	**–**	**–**
**41**	**–**	**–**	NA	NA	**–**	**–**	**–**	**–**	**–**	**–**	**–**	**–**
**42**	**–**	**–**	**–**	**–**	NA	NA	**–**	**–**	**–**	**–**	NA	NA
**43**	**–**	** + **	** + **	** + **	NA	NA	**–**	**–**	** + **	** + **	**–**	**–**
**44**	** + **	**–**	NA	NA	** + **	** + **	**–**	**–**	NA	NA	**–**	**–**
**45**	** + **	** + **	**–**	**–**	NA	NA	**–**	**–**	**–**	**–**	NA	NA
**46**	**–**	**–**	**–**	**–**	NA	NA	**–**	**–**	**–**	**–**	NA	NA
**47**	** + **	** + **	NA	NA	**–**	**–**	NA	NA	NA	NA	**–**	**–**
**48**	** + **	** + **	NA	NA	NA	NA	**–**	**–**	**–**	**–**	NA	NA
**49**	** + **	** + **	** + **	** + **	NA	NA	**–**	**–**	**–**	**–**	NA	NA
**50**	** + **	** + **	** + **	** + **	NA	NA	**–**	**–**	**–**	**–**	**–**	**–**
**51**	** + **	** + **	NA	NA	** + **	** + **	**–**	**–**	NA	NA	**–**	**–**
**52**	** + **	** + **	NA	NA	** + **	** + **	**–**	**–**	**–**	**–**	**–**	**–**
**53**	**–**	**–**	**–**	**–**	NA	NA	**–**	**–**	**–**	**–**	**–**	**–**
**54**	** + **	** + **	NA	NA	** + **	** + **	**–**	**–**	NA	NA	**–**	**–**
**55**	** + **	** + **	NA	NA	** + **	** + **	NA	NA	NA	NA	**–**	**–**
**56**	** + **	** + **	NA	NA	** + **	** + **	**–**	**–**	** + **	** + **	NA	NA

+ positive, *–* negative, *FLAIR* fluid-attenuated inversion recovery, *L* left eye, *NA* not available, *R* right eye, *SWI* susceptibility-weighted imaging, *T1w* T1-weighted, *T2w* T2-weighted, T2*w T2*-weighted

The interval between admission to hospital and MRI ranged from 0 days to 12 days (mean 2  days±2 days, median 1 day). In 53 of 57 cases (93.0%), MRI was performed within 5 days after admission. The interval between MRI and fundoscopy ranged from 0 days to 10 days (mean 2  days ± 2 days, median 1.5 days). In 41 of 45 cases (91.1%), the examinations were performed within a maximum interval of 5 days between each other. MRI was conducted after fundoscopy in 19 cases, prior to fundoscopy in 8 cases, and on the same day in 18 cases. In 11 cases, the exact date of the fundoscopy could not be ascertained.

**Fig. 1:**
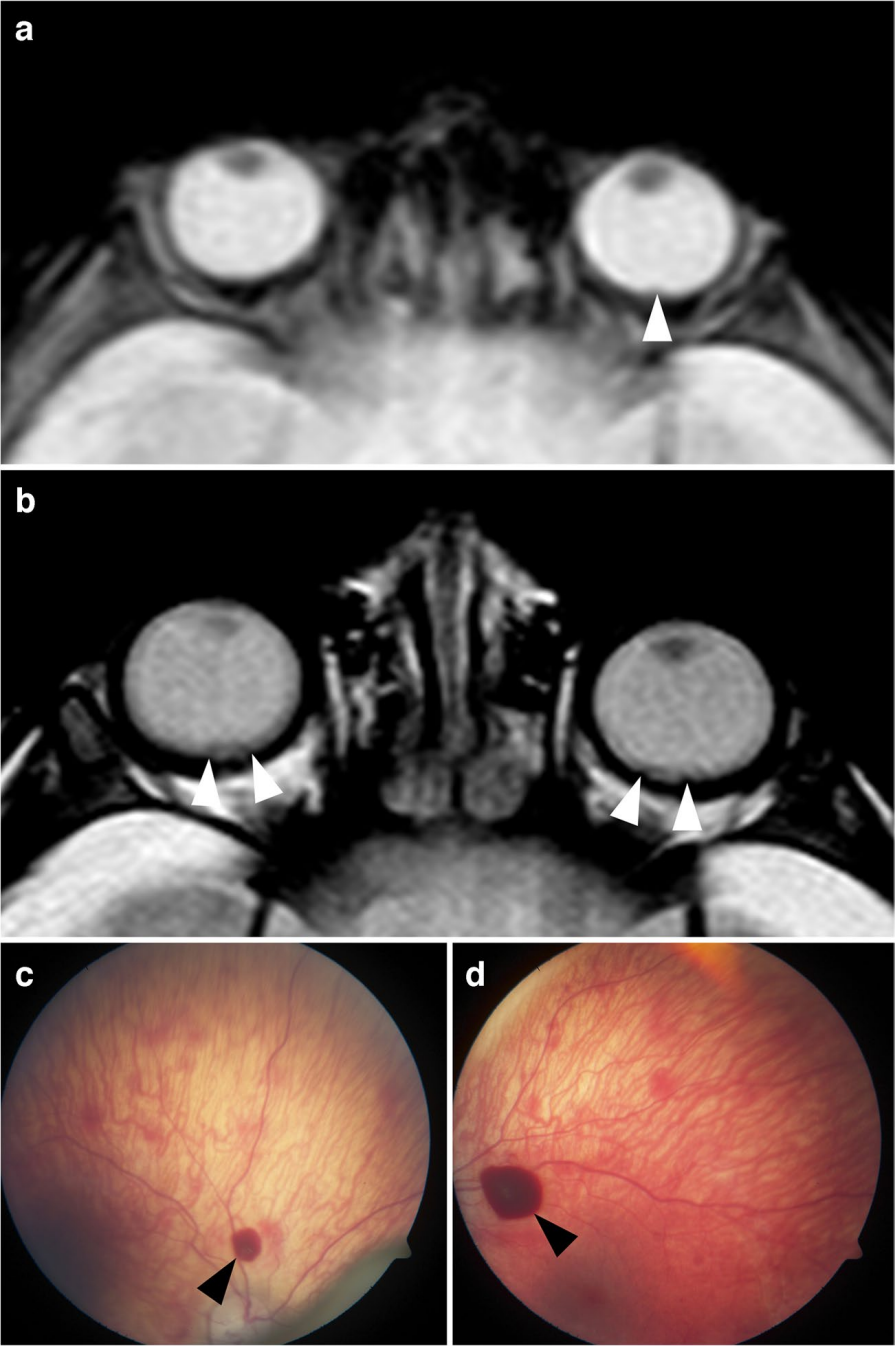
A 5-month-old girl with abusive head trauma following shaking and bilateral retinal hemorrhages detected in magnetic resonance imaging as well as in fundoscopy, both conducted on the same day.** a** Axial T2*-weighted magnetic resonance image through the orbits demonstrates a small retinal hemorrhage at the posterior pole of the left eye (*arrowhead*). **b** Axial T2-weighted image through the orbits shows irregularities at both posterior poles consistent with retinal hemorrhages (*arrowheads*). **c**, **d** Fundoscopy images of both eyes show delicate, diffuse subretinal hemorrhages across the entire fundi. Additionally, disc-sized epiretinal hemorrhages are visible along the major vascular arcades (*arrowheads*)

**Fig. 2:**
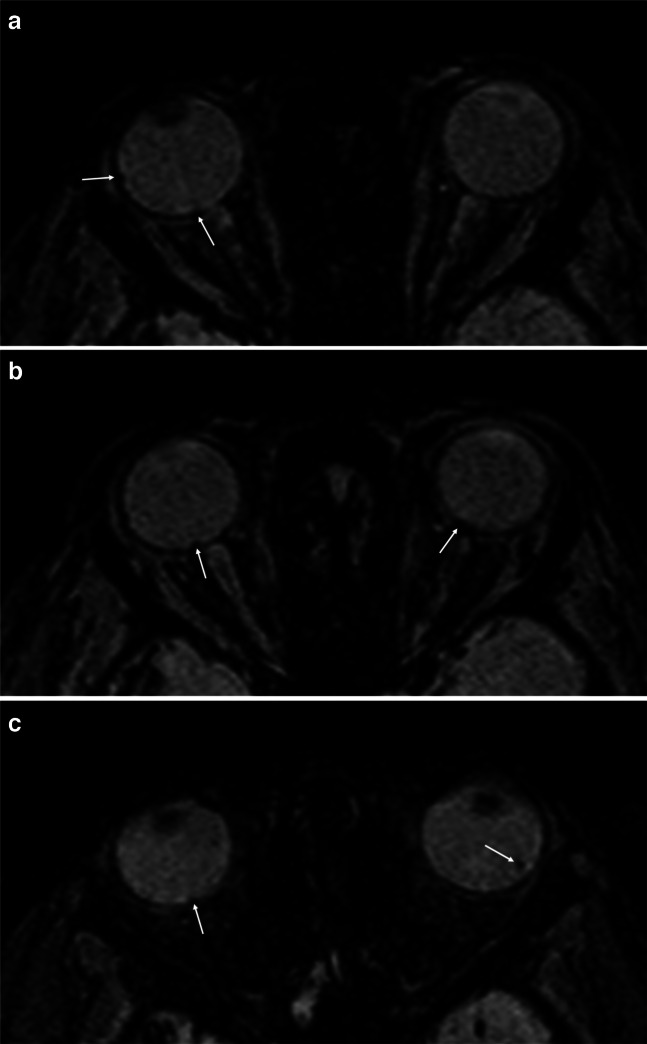
An 8-month-old boy with abusive head trauma following shaking and right-sided retinal hemorrhages detected on fundoscopy. In the right eye, pronounced streak-like epi- and subretinal hemorrhages were observed. The left eye showed central retinal edema without evidence of hemorrhage. **a**, **b**, **c** Axial susceptibility-weighted magnetic resonance images through different orbital planes demonstrate small signal voids within the retina at the posterior poles of both eyes and additionally at the lateral poles of both eyes (*arrows*)

Using fundoscopy as the reference standard, marked variability in sensitivities, specificity, positive predictive value, negative predictive value, positive likelihood ratio, and negative likelihood ratio for retinal hemorrhages was observed across the different MRI sequences (Table 3). SWI demonstrated the highest sensitivity (76.9%), followed by T2*w imaging (47.8%) (exemplified in Figs. 1 and 2). In contrast, morphological sequences (T2w, FLAIR, and T1w) showed substantially lower sensitivities (30.3%, 4.3%, and 1.5%). Again, comparison between “confession” and “non-confession” cases revealed no statistically significant differences in findings across any of the evaluated sequences.

**Table 3:** Overview of sensitivity, specificity, positive predictive value (*PPV*), negative predictive value (*NPV*), positive likelihood ratio (*LR+*), and negative likelihood ratio (*LR–*) for detection of retinal hemorrhages on different magnetic resonance imaging sequences

Imaging	Sensitivity (95% CI)	Specificity (95% CI)	PPV	NPV	LR+	LR–
T2*w	47.8% (32.9–63.1%)	88.5% (69.8–97.6%)	88.0%	48.9%	4.14	0.59
SWI	76.9% (56.4–91.0%)	87.5% (47.3–99.7%)	95.2%	53.8%	6.15	0.26
T1w	1.5% (0.0–8.3%)	100.0% (85.2–100%)	100%	26.4%	NA	0.98
T2w	30.3% (19.6–42.9%)	92.9% (76.5–99.1%)	90.9%	36.1%	4.24	0.75
FLAIR	4.3% (0.5–14.8%)	95.8% (78.9–99.9%)	66.7%	34.3%	1.04	1.00

*CI* confidence interval, *FLAIR* fluid-attenuated inversion recovery, *NA* not available, *SWI* susceptibility-weighted imaging, *T1w* T1-weighted, *T2w* T2-weighted, T2*w T2*-weighted

**Fig. 3:**
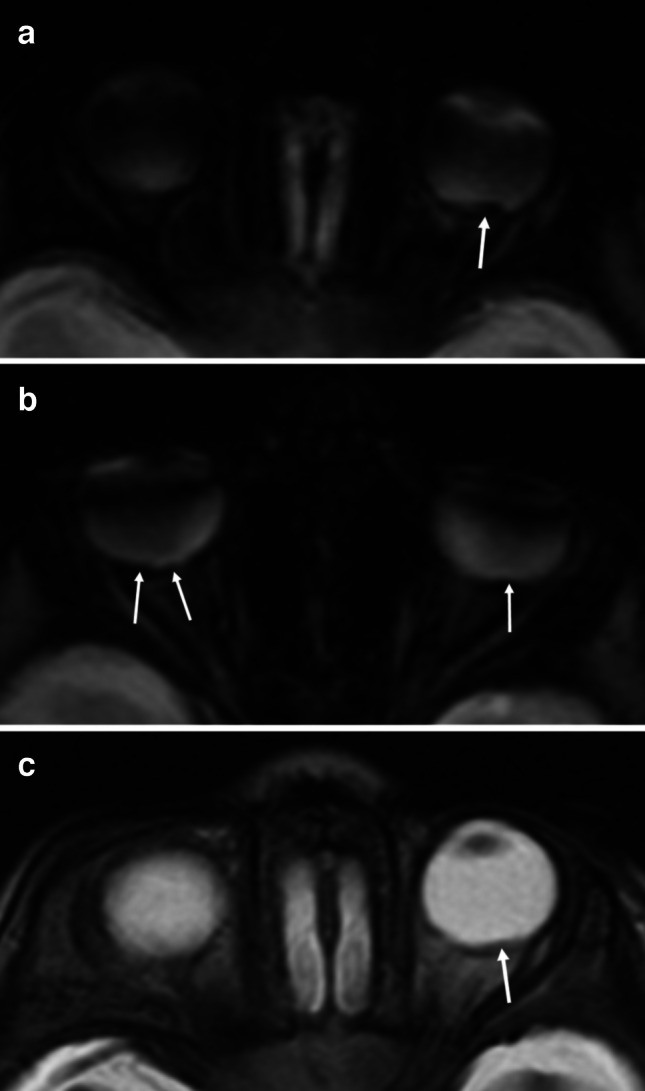
5-month-old girl with abusive head trauma following shaking and without retinal hemorrhages on fundoscopy. **a**, **b** Axial T2*-weighted magnetic resonance images through different orbital planes show subtle signal changes along the retina consistent with bilateral retinal hemorrhages (*arrows*). **c** Axial T2-weighted magnetic resonance image through the orbits demonstrates an abnormality at the dorsal pole of the left retina compatible with retinal hemorrhages (*arrows*)

**Fig. 4:**
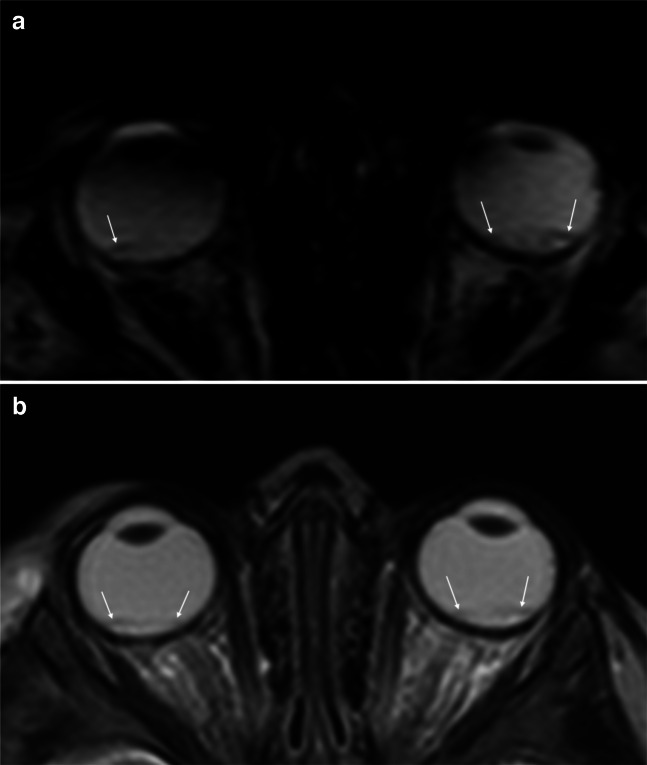
Six-month-old boy with abusive head trauma following shaking and left-sided retinal hemorrhages detected on fundoscopy. Ophthalmological assessment revealed localized hemorrhages in the left eye, with no hemorrhage detected in the right retina. **a** T2*-weighted magnetic resonance image and **b** T2-weighted magnetic resonance image show a distinct laminar elevation at the posterior pole of the retina in both eyes, highly consistent with retinal hemorrhages (arrows)

Regarding positive MRI findings where fundoscopy serving as reference standard was negative: In three cases (cases 31, 43, and 44), retinal hemorrhages were clearly identified on MRI despite negative or inconclusive findings on fundoscopy (exemplified in Figs. 3 and 4). In one case (case 31), which showed a negative fundoscopy, positive findings were detected in both eyes on T2*w imaging and in one eye on T2w and FLAIR imaging. In two additional cases (cases 43 and 44), in which fundoscopy identified retinal hemorrhages in only one eye, MRI revealed positive findings also in the contralateral eye on T2*w imaging and T2w imaging in one case (case 43) and on SWI in the other case (case 44).

In addition, a diagnostic discrepancy was observed within the MRI itself. In two cases (cases 11 and 29), the second most sensitive sequence (T2*w) failed to detect retinal hemorrhages in one eye, whereas retinal hemorrhages were visible on the less sensitive T2w sequence.

## Discussion

The diagnosis of AHT remains challenging, as clinical findings are often non-specific and caregiver-reported histories are frequently absent, insufficient, or inconsistent. It is therefore all the more important to improve the detection and assessment of potential signs of AHT. Retinal hemorrhages represent a common and significant finding in cases of AHT following shaking, most likely resulting from traction and shearing forces exerted on the eyeball via the optic nerve. Although retinal hemorrhages are considered best detectable by fundoscopy, they can often be visualized effectively on MRI studies, which are routinely performed for diagnostic evaluation of the brain. Given the limitations and constraints of fundoscopy—such as limited availability of pediatric ophthalmologists, patient instability, or the need for sedation—the depiction of retinal hemorrhages on MRI may represent an additional tool in the diagnostic process as well as an objective documentation method.

This study demonstrated that MRI, particularly using specialized GRE sequences, allows for the visualization of retinal hemorrhages in cases of AHT following shaking to a reasonable extent. The highest sensitivity (76.9%) was achieved using SWI consistent with findings by Zuccoli et al. [16], who reported a sensitivity of 75%. Other studies observed distinct lower sensitivities of 50.0% and 57.6% [18, 19]. A similar sensitivity was achieved in our study with T2*w imaging (47.8%), which is generally inferior to SWI in detecting microbleeds. Thus, T2*w imaging appears to be a practical alternative in settings where SWI is unavailable.

The differences in SWI sensitivities across studies may be in part attributable to differences in the severity of retinal hemorrhages included [27]. In addition, variable intervals between fundoscopy and MRI may have allowed partial or near-complete resorption of retinal hemorrhages in some cases [15]. While the SWI subgroup in our study (*n*=17) was relatively small compared to studies reporting lower SWI sensitivities (up to *n*=23) [17–19], the study by Zuccoli et al. describing sensitivities in SWI comparable to our study included a cohort of at least 28 participants [16]. Table 4 provides an overview and comparison of these studies [16–19] with our data.

**Table 4:** Comparison of neuroimaging studies on retinal hemorrhages in pediatric abusive head trauma (*AHT*)

	**Zuccoli et al. (2013) **[16]	**Gencturk et al. (2019) **[17]	**Tamburaj et al. (2019) **[18]	**Gencturk et al. (2021) **[19]	**Present study**
**Total *n***	28	55, AHT subgroup: 16	26	67, AHT subgroup: 23	57, cases with MRI and available fundoscopy: 56
**Cohort**	Suspected AHT	• Non-AHT (*n*=35/55) • AHT (*n*=16/55) • Indeterminate (*n*=4/55)	AHT	• AHT (*n*=23/67) • Non-AHT (*n*=38/67) • Indeterminate group (*n*=6/67)	AHT following shaking
**Diagnosis of AHT based on**	NA	Legal conviction or confession	Child protection service physicians in collaboration with a multidisciplinary team	Child abuse team and available legal assessment	Medico-legal expert opinion, partially confirmed by confession
**Age**	Mean 10.9 months±7.2 months	Mean 11.15 months±9.77 months	Range 1.6 months to 48 months, mean 9.1	Mean 12.6 months±11.8 months	Range 0–17 months, mean 4.1 months ± 3.7 months
**Sequences**	SWI, SWI high-resolution of orbits	SWI	SWI	SWI, T2	T2*, SWI, T1, T2, FLAIR
**Retinal hemorrhages in fundoscopy**	*n*=21/28 (75%)	*n*=11/16 (69%)	R: *n*=18/21 (85.7%) L: *n*=16/21 (76.2%)	Each eye considered separately: *n*=33/46 (71.7%)	Each eye considered separately: *n*=80/112 (71.4%)
**Retinal hemorrhages in MRI**					
High-Res SWI	NA	-	-	-	-
SWI	NA	*n*=7/16 (44%)	R: *n*=9/26 (34.6%) L: *n*=8/26 (30.8%)	*n*=19/46 (41.3%)	*n*=21/34 (61.8%)
T2*	-	-	-	-	*n*=25/72 (34.7%)
T1	-	-	-	-	*n*=1/88 (1.1%)
T2	-	-	-	*n*=9/46 (19.6%)	*n*=22/94 (23.4%)
FLAIR	-	-	-	-	*n*=3/70 (4.3%)
**Time gap between fundoscopy and MRI**	• DFE prior to the MRI, *n*=5 • Both exams on the same day, *n*=5 • MRI after DFE, *n*=18	NA	Range from 0–7 days in *n* = 19/21	Mean interval between DFE and MRI 1.56 days±1.07 days (range 0–5 days)^a^	Range from 0–10 days (mean 2 days±2 days), in 91.1% ≤5 days (*n*=41/45)
**Sensitivity (%)**					
High-Res SWI	83%	-	-	-	-
SWI	75%	NA	50%	57.6%^b^	76.9%
T2*	-	-	-	-	47.8%
T1	-	-	-	-	1.5%
T2	-	-	-	27.3%^b^	30.3%
FLAIR	-	-	-	-	4.3%
**Specificity**					
High-Res SWI	100%	-	-	-	-
SWI	100%	NA	100%	100%	87.5%
T2	-	-	-	100%	88.5%

*%* Percent, - not done, *DFE* dilated fundus examination, *FLAIR* fluid-attenuated inversion recovery imaging, *High-Res SWI* high-resolution SWI, *L* left eye, *MRI* magnetic resonance imaging, *n* number of cases, *NA* not available, *Non-AHT* nonabusive head trauma, *R* right eye, *SWI* susceptibility-weighted imaging, *T1* T1-weighted imaging, *T2* T2-weighted imaging, T2* T2*-weighted imaging, ^a^The information comprises for an overall cohort (*n*=67) consisting of AHT, non-AHT and indeterminate group, ^b^Based on our own calculations

A sensitivity of around 75% indicates that SWI—and, to a lesser extent, T2*w imaging—is a highly useful technique for visualizing retinal hemorrhages in AHT following shaking. Zuccoli et al. [16] even reported sensitivities of up to 83% using high-resolution SWI of the orbit. This sequence can be considered an excellent alternative, particularly in children who are difficult to examine by fundoscopy. However, the extended acquisition time required for this sequence remains a substantial limiting factor for its implementation in routine clinical practice. Additionally, although SWI is more sensitive than T2*w imaging*,* its greater vulnerability to motion artifacts supports consideration of acquiring both sequences to help ensure interpretable imaging. Nevertheless, ongoing advances in accelerated imaging techniques may further improve the feasibility of incorporating such sequences into standard diagnostic protocols in the future.

In our study, T2w imaging showed a markedly lower sensitivity (30.3%) than GRE imaging. This was similar to the 27.3% reported by Gencturk et al. [19]. Notably, in our cohort, retinal hemorrhages were observed in two cases (pertaining to one eye) on T2w imaging but not on T2*w imaging. This discrepancy may be due to differences in spatial resolution or slice thickness, which could limit the ability of T2*w imaging to detect such lesions. It is also conceivable that certain lesion types, such as older hemorrhagic lesions or post-traumatic proliferative vitreoretinopathy, are more readily identifiable or more precisely delineated on T2w sequences, as previously suggested by other authors [28]. Overall, these observations suggest that T2w imaging retains diagnostic value in assessing retinal hemorrhages or related lesions in AHT.

Regarding the diagnostic value of other morphological sequences we observed very low sensitivities of 1.5% on T1w and 4.3% on FLAIR imaging. No directly comparable AHT studies are available for these sequences. However, Beavers et al. [27] correlated MRI detection of retinal hemorrhages with different grades or severities on fundoscopy in a mixed cohort of trauma patients (“accidental,” “non-accidental,” and “brain trauma”) aged ≤2 years (*n*=77). They reported somewhat higher sensitivities: 26% for FLAIR imaging and 18% for T1w imaging. A comparison is difficult because of the heterogeneous mixture of different trauma cases of unknown proportions and the low frequencies of retinal hemorrhages detected on fundoscopy (60%, 46/77). Our study and other AHT studies consistently show detection rates exceeding 69% on fundoscopy [17–19, 29]. Of note, in the Beavers et al. study [27], every case that was positive on T1w imaging and FLAIR imaging showed a very high grade or severity of retinal hemorrhages on fundoscopy. Consequently, the current knowledge does not indicate that T1w or FLAIR imaging provides useful additional information for detecting retinal hemorrhages in AHT.

In contrast to previous studies, our results revealed clear MRI evidence of retinal hemorrhages (partly pertaining one eye and partly both eyes) that were not found by ophthalmologists in corresponding fundoscopy. These were not considered false positives, as MRI revealed clear abnormalities compatible with retinal hemorrhages. In these three cases, fundoscopy was performed in close temporal proximity to MRI: on the same day in two cases and 2 days after MRI in the third case. Thus, resolution of the retinal hemorrhages cannot account for the differing findings. This demonstrates that MRI may detect retinal hemorrhages while fundoscopy can miss them, whether due to limitations in timing, technical quality, or clinical feasibility. This underscores the potential of MRI to improve the evaluation of suspected AHT cases, even though it cannot, like fundoscopy, identify high-risk retinal hemorrhage features associated with AHT (e.g., multilayer involvement, ora serrata extension, or retinal folds), which are important for the differential diagnosis of retinal hemorrhages related to other conditions, including accidental trauma, hematologic disorders, or infection.

Several limitations of this study must be considered. Foremost is the heterogeneity of the imaging dataset. Conducted as a multicenter investigation over a 10-year period, MRI scans were acquired using diverse protocols, scanner types, field strengths, and sequence parameters across multiple institutions. This lack of standardization likely influenced both the sensitivity and comparability of the sequences, particularly for subtle findings such as retinal hemorrhages. While the multicenter design introduces some heterogeneity, it also strengthens external validity and reflects real-world clinical practice.

A further key limitation of our study is the potential temporal gap between MRI and fundoscopy, so that retinal hemorrhages may partially or fully resolve in the meantime [15]. Consequently, discrepancies between MRI and fundoscopy may reflect differences in examination timing rather than true variations in diagnostic sensitivity. In addition, fundoscopy is not always performed under standardized conditions or documented with photographic evidence, limiting the objectivity and reproducibility of the fundoscopy findings used for comparison. In our study, MRI was performed shortly after hospital admission (93.0% of cases within ≤5 days), which is assumed to be temporally close to the traumatic event and thus enhanced the likelihood of detecting retinal hemorrhages in general [15].

Another important limitation is that we assessed only the presence of retinal hemorrhages on fundoscopy without evaluating the extent of these findings, which is an important aspect of this examination and may be relevant for differential diagnosis.

The inclusion criteria of our study also introduce potential selection bias. Only children who arrived alive at the hospital were included, a necessity for comprehensive clinical and medico-legal documentation. However, this approach likely underrepresents more severe or fatal AHT cases that may exhibit more extensive retinal hemorrhages, as documented in postmortem studies. Consequently, the true prevalence of MRI-detectable retinal hemorrhages in AHT following violent shaking may be underestimated.

It is also important to recognize that not all retinal hemorrhages visible on MRI are specific to AHT. Retinal hemorrhages can also occur in neonates in the context of birth-related trauma, but birth-related retinal hemorrhages are no longer detectable within approximately 4 weeks after birth [30–32]. In our cohort, only one child was younger than 1 month; as both the fundoscopy and MRI findings were negative, this case was therefore not excluded from the study cohort on this bias.

Despite these limitations, the present study has several notable strengths. It represents one of the largest and most comprehensively documented cohorts of AHT examined for retinal hemorrhages using MRI. All cases were clinically and medico-legally well documented and, to the best of our knowledge, can be attributed to a sole trauma mechanism: violent shaking. This enhances diagnostic reliability and minimizes bias by other trauma mechanisms where retinal hemorrhages are occurring less frequently, e.g., blunt force against the neurocranium. Furthermore, the inclusion of both “confession” and “non-confession” cases reduces the potential of circular diagnostic bias, which can affect studies on AHT.

Looking ahead, prospective studies may scrutinize these findings using standardized retinal imaging tools such as RetCam, assess interobserver agreement for both MRI and fundoscopy to further support reliability, and apply standardized orbital imaging protocols with near-simultaneous fundoscopy correlation. Incorporating advanced imaging techniques and automated image-analysis tools—including artificial intelligence—may further improve the sensitivity, reproducibility, and objectivity of retinal hemorrhages detection on MRI.

In conclusion, this study demonstrates that retinal hemorrhages in AHT following shaking can be detected on MRI with good sensitivity, particularly using SWI and T2*w sequences, while morphological T2w imaging can add complementary value. Although MRI cannot replace fundoscopy, systematic assessment of the retina on these sequences can be a supplementary diagnostic tool in suspected AHT cases.

## Data Availability

The datasets generated during and/or analysed during the current study are available from the corresponding author on reasonable request.
